# Combination chemotherapy with epirubicin, docetaxel and cisplatin (EDP) in metastatic or recurrent, unresectable gastric cancer

**DOI:** 10.1038/sj.bjc.6601891

**Published:** 2004-06-08

**Authors:** S-H Lee, W K Kang, J Park, H Y Kim, J H Kim, S I Lee, J O Park, K Kim, C W Jung, Y S Park, Y-H Im, M H Lee, K Park

**Affiliations:** 1 Division of Hematology and Oncology, Department of Medicine, Samsung Medical Center, Sungkyunkwan University School of Medicine, 50 Ilwon-dong, Kangnam-gu, Seoul 135-710, Korea

**Keywords:** chemotherapy, cisplatin, docetaxel, epirubicin, gastric carcinoma

## Abstract

Based on single agent activities and the additive or synergistic effects of three individual drugs in gastric cancer, we performed a phase II study of a new regimen combining epirubicin, docetaxel and cisplatin (EDP) for unresectable gastric cancer. The patients with histologically confirmed metastatic or recurrent, unresectable gastric cancer and no history of palliative chemotherapy were eligible for this trial. In total, 40 mg m^−2^ epirubicin (reduced to 30 mg m^−2^ due to high incidence of febrile neutropaenia; 75%) intravenously (i.v.) over 30 min, followed by 60 mg m^−2^ docetaxel i.v. over 1 h, then 75 mg m^−2^ cisplatin i.v. over 1 h was administered every 3 weeks. Between January 2002 and February 2003, 30 patients (epirubicin 40 mg m^−2^, eight; 30 mg m^−2^, 22) were enrolled. The median age was 52 years (range, 33–68). The patients received a median of four cycles (range, 1–8). One patient (3%) achieved a complete response, 13 (43%) showed partial responses, 13 (43%) had stable diseases and three (10%) progressed. The overall response rate was 47% (95% CI, 28–66%), and the median duration of response was 5.0 months (95% CI, 3.0–7.0). The median time to progression was 4.1 months (95% CI, 2.4–5.9), and the median overall survival was 11.0 months (95% CI, 9.5–12.4). Grade 4 neutropaenia were observed in 41%, and febrile neutropaenia in 32%, out of the patients receiving 30 mg m^−2^ of epirubicin. Grade 3 nonhaematological toxicities included nausea, vomiting, anorexia and peripheral neuropathy. In conclusion, EDP is active in gastric cancer, with a manageable and predictable toxicity profile.

Gastric cancer is the most common malignancy in Korea (Bae *et al*, 2002). The prognosis of unresectable gastric cancer has been improved by cytotoxic chemotherapy, but median survival rarely exceeds 1 year (Pyrhonen *et al*, 1995; Glimelius *et al*, 1997; Webb *et al*, 1997; Ohtsu *et al*, 2003).

Single agents such as doxorubicin, cisplatin, 5-fluorouracil (5-FU) and mitomycin-C have modest activities, showing response rates of about 20% against gastric cancer. The median duration of response is usually between 3 and 4 months, and occasional complete responses (CRs) have been noted (Moertel and Lavin, 1979; Cocconi *et al*, 1982; Aabo *et al*, 1985).

Many combinations of cytotoxic chemotherapeutic agents have been developed to improve the remission rate and duration of survival. In the late 1980s and early 1990s, FAM (5-FU, doxorubicin, mitomycin-C), FP (5-FU, cisplatin), FAMTX (5-FU, doxorubicin, methotrexate), EAP (etoposide, doxorubicin, cisplatin) and ECF (epirubicin, cisplatin, protracted 5-FU) showed high response rates in phase II trials, but lower response rates and overall survival (OS) of less than 1 year in randomised trials (Wilke and Cutsem, 2003). Randomised trials showed FAMTX was superior to FAM (in efficacy) and EAP (in safety), and ECF was superior to FAMTX in terms of response rate and survival (Wils *et al*, 1991; Kelsen *et al*, 1992; Webb *et al*, 1997). However, the median survival of the ECF regimen ranged from 8 to 10 months in the phase III trials (Webb *et al*, 1997; Ross *et al*, 2002).

New agents such as taxane, irinotecan and oxaliplatin combined with old agents such as cisplatin and 5-fluorouracil are currently under evaluation to further improve treatment outcome (Wilke and Cutsem, 2003). Docetaxel as a single agent showed response rates of 17–24% (Sulkes *et al*, 1994; Einzig *et al*, 1996), and the combination of docetaxel and cisplatin has shown a response rate of 37–56% and OS of 9–10.4 months (Roth *et al*, 2000; Ridwelski *et al*, 2001).

Although the combination of docetaxel and anthracycline has not been studied enough in gastric cancer, the synergism between docetaxel and anthracycline is well established in other cancers, especially breast cancer (Pagani *et al*, 2000). Compared with doxorubicin, epirubicin has the advantages of less cardiotoxicity and less myelosuppression, with similar cytotoxic effect (Cersosimo and Hong, 1986; Vorobiof and Falkson, 1989). A combination of epirubicin and docetaxel showed a response rate of 22% and progression-free survival of 16 weeks when used as a second-line treatment in advanced gastric cancer (Andre *et al*, 1999). In addition, epirubicin has proven to have synergistic clinical effect with cisplatin (Kyoto Research Group for Chemotherapy of Gastric Cancer, 1992) in gastric cancer.

Combination chemotherapy with 40 mg m^−2^ of epirubicin, 75 mg m^−2^ of docetaxel and 75 mg m^−2^ of cisplatin were administered in transitional cell carcinoma (Pectasides *et al*, 2000). The study reported tolerable toxicity profiles, but 53% of the patients were required at least one dose reduction due to haematological toxicities. According to the findings of the study, we planned to administer 60 mg m^−2^ of docetaxel instead of 75 mg m^−2^ in combination with the other two drugs.

Based on the single agent activities of epirubicin, docetaxel and cisplatin (EDP), and the additive or synergistic clinical effects of these three drugs in gastric cancer, we performed a phase II trial of combination chemotherapy with EDP for patients with metastatic or recurrent, unresectable gastric cancer.

## MATERIALS AND METHODS

This was an open-label, single institution, phase II study of combination therapy with EDP in metastatic or recurrent, unresectable gastric cancer. Patients were enrolled at Samsung Medical Center between January 2002 and February 2003. The study was approved by the institutional review board, and written informed consent was obtained from each patient.

### Patient eligibility

Patients with histologically confirmed metastatic or recurrent, unresectable gastric adenocarcinoma were eligible for this study. Actually all patients had metastatic gastric adenocarcinoma, with or without history of surgery with curative intent. All patients were required to be between 18 and 70 years, to have an Eastern Cooperative Oncology Group (ECOG) performance status (PS) of 0–2, and to have bidimensionally measurable disease (defined as the presence of at least one index lesion capable of two-dimensional measurement by computed tomography scan or chest X-ray above 2 cm in greatest diameter). Any history of chemotherapy for palliation was not allowed, but adjuvant chemotherapy elapsing more than 12 months previously was allowed. Other eligibility criteria included an absolute neutrophil count (ANC) ⩾1500 mm^−3^, a platelet count ⩾100 000 mm^−3^, serum aspartate aminotransferase (AST) and alanine aminotransferase (ALT) activities ⩽3.0 times the upper normal limit (UNL) (in cases of liver metastasis, AST and ALT ⩽5.0 UNL), serum bilirubin level ⩽1.25 times UNL, creatinine clearance (Cockroft formula) ⩾60 ml min^−1^ and left ventricular ejection fraction ⩾50%. Patients with metastasis to the central nervous system were excluded from the study. Prior history of another malignancy within 5 years of study entry, apart from basal cell carcinoma of the skin or carcinoma *in situ* of the uterine cervix, or grade 2–4 peripheral neuropathy, precluded participation in the current trial. Patients with clinically significant cardiac disease as defined by symptomatic ventricular arrhythmias, history of congestive heart failure, or history of previous myocardial infarction within 12 months of study entry were also excluded.

## STUDY PROTOCOL

Patients received 40 mg m^−2^ (reduced to 30 mg m^−2^ due to unexpectedly high incidence of febrile neutropaenia; 75%) of epirubicin intravenously (i.v.) over 30 min, followed by 60 mg m^−2^ of docetaxel i.v. over 1 h, and then followed by 75 mg m^−2^ of cisplatin i.v. over 1 h on day 1. Cycles were repeated every 3 weeks. Dexamethasone premedication was used for prophylaxis of docetaxel-induced hypersensitivity and fluid retention. Adequate hydration and intravenous mannitol were used for the prophylaxis of cisplatin-induced nephrotoxicity. Half saline 1000 ml plus KCl 20 mEq was administered intravenously over 2 h each before and after cisplatin. Patients were discontinued from the study therapy when there was evidence of disease progression, the patient experienced unacceptable toxicity, the patient requested discontinuation, or the investigator decided that the patient should be withdrawn.

The primary end point of the trial was response rate. The secondary end points were duration of response, time to progression (TTP), OS and toxicity.

### Dose modification

Chemotherapy was withheld if the ANC was <1500 mm^−3^ or the platelet count was <100 000 mm^−3^ on day 1. In this case, the complete blood cell count was repeated at least weekly and chemotherapy was restarted as soon as the ANC reached ⩾1500 mm^−3^ and platelet count ⩾100 000 mm^−3^. If nadir ANC was less than 500 mm^−3^ or the nadir platelet count was less than 50 000 mm^−3^, the doses of epirubicin and docetaxel were reduced to 30 and 45 mg m^−2^, respectively, and in the patients who received 30 mg m^−2^ of epirubicin, docetaxel alone was reduced to 45 mg m^−2^. If the calculated creatinine clearance (Ccr) was less than 50 ml min^−1^, treatment was delayed, and serum creatinine was measured weekly. Treatment was resumed if calculated Ccr increased above 50 ml min^−1^; if this increase was not achieved by day 35, the patient was taken off study. If the calculated Ccr was 50–59 ml min^−1^ at any time, cisplatin was administered with the dose of 60 mg m^−2^. If grade 3–4 neurotoxicity or ototoxicity occurred, cisplatin was withheld in the subsequent cycles. If grade 3–4 nausea or vomiting was not controlled with adequate management, cisplatin was reduced to 60 mg m^−2^. If grade 3–4 nonhaematologic toxicity other than alopecia and those above mentioned, the treatment was withheld until recovery to grade 0 or 1 and the doses of epirubicin and docetaxel were reduced to 30 and 45 mg m^−2^, respectively, and in the patients who received 30 mg m^−2^ of epirubicin, docetaxel alone was reduced to 45 mg m^−2^. If patients required a delay of longer than 2 weeks, they were removed from the study.

### Assessment of efficacy and toxicity

At study entry, the following investigations were performed: full history taking and physical examination, complete blood cell count (CBC), chemistry, chest X-ray, echocardiography and computed tomography scan. All investigations except echocardiography were repeated before every cycle. Computed tomography scans were performed optimally to document disease extent and to evaluate response to treatment, every two cycles and when needed for the confirmation of response and suspected disease progression. CBC was repeated every week during the first cycle and the following cycle if the patient experienced grade 4 haematologic toxicity. Otherwise, CBC was repeated every 3 weeks.

Complete response was defined as the complete disappearance of all clinically detectable disease for at least 4 weeks. Partial response (PR) was defined as a more than 50% decrease in the sum of the products of the two longest perpendicular diameters of all measurable lesions for at least 4 weeks with no increase in size of any area of known malignant disease and no appearance of new areas of malignant disease. Progressive disease (PD) was defined as a greater than 25% increase in the sum of the products of the perpendicular diameters of all measurable lesions or the appearance of any new lesion. All other outcomes were scored as stable disease (SD).

Response rate was calculated as the ratio of number of patients who achieved CR or PR to the number of intent-to-treat (ITT) patients.

Duration of response was calculated from the first day of treatment to the date on which PD was first observed or of the last follow-up, for the group of responding patients. Time to progression was calculated from the first day of treatment to the date on which PD was first observed or of the last follow-up. Overall survival was calculated from the first day of treatment to the date of death or last follow-up.

Toxicity was assessed according to the National Cancer Institute common toxicity criteria (NCI-CTC) scale version 2.0. The severity of any toxicities not defined in the NCI-CTC were graded as 1=mild, 2=moderate, 3=severe or 4=very severe.

### Statistical analysis

Descriptive statistics were reported as proportions and medians. Kaplan–Meier estimates were used in the analysis of all time-to-event variables, and the 95% confidence interval (CI) for the median time to event was computed. The dose intensity (DI) was calculated as the ratio of the total dose (expressed in milligrams) per square metre of the patient, divided by the total treatment duration expressed in days. In this calculation, the end of treatment was considered to be 21 days after day 1 of the last cycle of chemotherapy. The relative DI was calculated as the ratio of the DI actually delivered to the DI planned by the protocol.

According to Simon's two-stage optimal design, a sample size of 25 was required to accept the hypothesis that the true response rate is greater than 45 with 80% power, and to reject the hypothesis that the response rate is less than 20 with 5% significance. At the first stage, if there were fewer than two responses out of the initial 15 patients, the study would terminate. Although the target number of patients was 25, we planned to recruit 20% more than the target number of patients considering drop-out.

SPSS for Windows (SPSS Inc., Chicago, IL, USA) was used for statistical analysis.

## RESULTS

### Patient characteristics

From January 2002 to February 2003, 30 patients were enrolled. The clinical characteristics of the enrolled patients are provided in Table 1

**Table 1:** Patient characteristics

**Characteristic**	**Number of patients**
Eligible/total patients	30/30
Age (years: median (range))	52 (33–68)
Male/female	25/5
Performance status: ECOG 0/1	3/27
	
*Grade*	
Moderately differentiated	7
Poorly differentiated	21
	
*Predominant metastatic sites*	
Liver	11
Lymph node	15
Peritoneum	1
Others	3
	
*Previous treatment*	
Surgery with curative intent	4
Palliative surgery	9
Gastrectomy	8
Metastasectomy	2
Adjuvant chemoradiation	1

. All patients had gastric adenocarcinoma. It did not include tumours of gastroesophageal junction. The median age was 52 years (range, 33–68), and there were 25 (83%) men and five women. Four patients received surgery with curative intent, with a disease-free interval of 9–32 months. Another nine patients received palliative debulking surgery 1–2 months before the study. The major involved organs were liver and intra-abdominal lymph nodes.

### Delivery of drug

The patients received a median of four (range, 1–8) cycles. The average relative dose-intensity was 0.92 for epirubicin, 0.86 for docetaxel and 0.90 for cisplatin in the patients who received 30 mg m^−2^ epirubicin. Dose reduction was required in eight patients (eight cycles) and treatment was delayed in seven patients (nine cycles) out of 22 patients who received 30 mg m^−2^ epirubicin.

### Efficacy

All 30 patients were evaluable for response. We observed one CR, 13 PRs and 13 SDs (Table 2

**Table 2:** Response to combination chemotherapy

**Response**	**Total (*N*=30)**	**Epi 30 mg m^−2^ (*N*=22)**	**Epi 40 mg m^−2^ (*N*=8)**
Complete response	1 (3%)	1 (5%)	0
Partial response	13 (43%)	9 (41%)	4 (50%)
Stable disease	13 (43%)	9 (41%)	4 (50%)
Progressive disease	3 (10%)	3 (14%)	0

**Figure 1:**
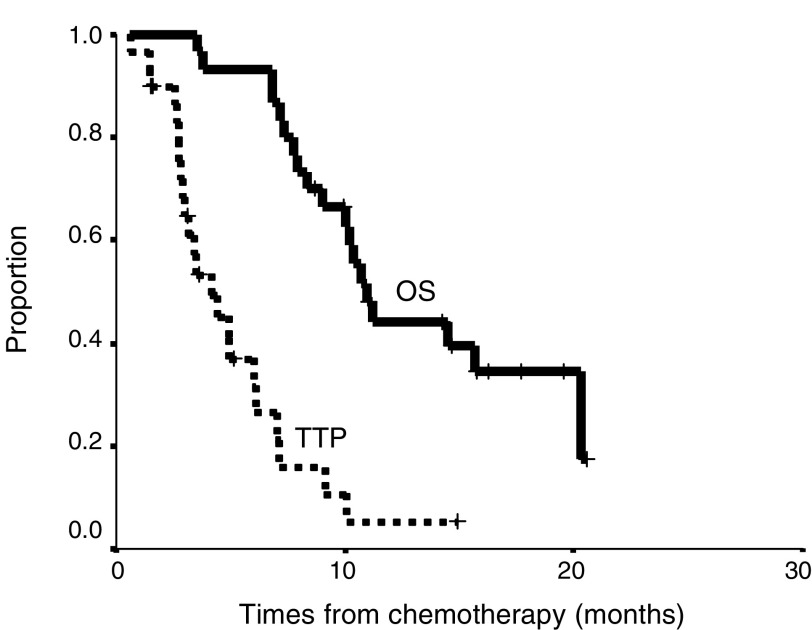
Overall survival (OS) and time to progression (TTP) curve.

Epi=epirubicin.

). The response rate was 47% (95% CI, 28–66%), and the median duration of response was 5.0 months (95% CI, 3.0–7.0) for all responders. The median follow-up time was 17.7 months. The median TTP was 4.1 months (95% CI, 2.4–5.9), and the median OS was 11.0 months (95% CI, 9.5–12.4) (Figure 1

).

### Toxicity

Table 3

**Table 3:** Toxicity of combination chemotherapy

**Toxicity**	**Grade 1**	**Grade 2**	**Grade 3**	**Grade 4**
*Haematological toxicity (30 patients; maximal grade)*				
Leucopaenia	5 (17%)	6 (20%)	9 (30%)	7 (23%)
Neutropaenia	3 (10%)	2 (7%)	6 (20%)	16 (53%)
Anaemia	7 (23%)	18 (60%)	5 (17%)	—
Thrombocytopaenia	5 (17%)	3 (10%)	1 (3%)	—
				
*Nonhaematological toxicity (30 patients)*				
Fatigue	8 (27%)	1 (3%)	—	—
Anorexia	9 (30%)	1 (3%)	1 (3%)	—
Nausea	6 (20%)	6 (20%)	4 (13%)	—
Vomiting	4 (13%)	3 (10%)	1 (3%)	—
Constipation	4 (13%)	—	—	—
Diarrhoea	3 (10%)	3 (10%)	—	—
Stomatitis	6 (20%)	1 (3%)	—	—
Neuropathy	8 (27%)	—	1 (3%)	—

summarises the toxicity observations. Grade 4 neutropaenia was observed in 16 patients (53%). The patients who received 40 mg m^−2^ of epirubicin (EPI-40) experienced grade 4 neutropaenia more frequently than those receiving 30 mg m^−2^ of epirubicin (EPI-30) (88 *vs* 41%, *P*=0.039). Febrile neutropaenia was observed more frequently in EPI-40 than EPI-30 (75 *vs* 32%, *P*=0.034). Recurrent febrile neutropaenia was observed in four patients (one in EPI-30, three in EPI-40). No treatment-related mortality was observed.

Grade 3 nonhaematological toxicities were observed in some patients, including nausea, vomiting, anorexia and peripheral neuropathy. Grade 4 nonhaematological toxicity was not observed.

## DISCUSSION

We report the results of combination chemotherapy with EDP in metastatic gastric cancer patients. The response rate of 47% and median survival of 11.0 months in this study indicate that this regimen is active against gastric cancer and worthy of further investigation.

The high prevalence in Korea of unresectable gastric cancer, the poor prognosis and slow improvement of treatment outcomes prompted us to develop a new regimen with high activity. The addition of taxane to active drug lists enabled us to broaden the selection of treatment options. Docetaxel in combination with cisplatin (Roth *et al*, 2000; Ridwelski *et al*, 2001) or epirubicin (Andre *et al*, 1999) showed substantial response rates as first- and second-line chemotherapy in gastric cancer. An acceptable toxicity profile observed from experience in transitional cell carcinoma (Pectasides *et al*, 2000) encouraged us to investigate this triple combination in gastric cancer.

We frequently encounter a response rate of 47% in the setting of phase II studies in gastric cancer, but a median OS of 11.0 months is a favourable result. Although selection bias in phase II trials should be considered, median OS more than 10.0 months is not frequently reported. The well-proven regimen ECF reported high response rate of 71%, but median OS was 8.2 months in a phase II trial (Findlay *et al*, 1994), and 8.9 months and 9.4 months in phase III trials (Webb *et al*, 1997; Ross *et al*, 2002). An interim analysis of a large, randomised phase III trial comparing DCF and CF showed median OSs of 10.2 and 8.5 months, respectively (Ajani *et al*, 2003).

The initial protocol with 40 mg m^−2^ epirubicin resulted in an unexpectedly high incidence of grade 4 neutropaenia (seven out of eight patients) and febrile neutropaenia (six out of eight patients). Therefore, we decided to reduce the epirubicin dose, and the subsequent 22 patients received 30 mg m^−2^ of epirubicin as a starting dose. More patients experienced febrile neutropaenia than those with transitional cell carcinoma who received the similar regimen (Pectasides *et al*, 2000). The probable explanation is the less frequent use of haematopoietic growth factors in our study. In the previous study, the authors administered 75 mg m^−2^ docetaxel, 40 mg m^−2^ epirubicin and 75 mg m^−2^ cisplatin, and they used prophylactic haematopoietic growth factors with dose reduction in cases of grade 3–4 neutropaenia and/or febrile neutropaenia (Pectasides *et al*, 2000). However, we only reduced the dose in the subsequent cycles, without using prophylactic haematopoietic growth factor. We initially planned to include the patients with moderate liver function abnormality, that is, AST/ALT <5 times the UNL in case of liver metastasis. We speculated whether the generous inclusion criteria for liver function abnormality were responsible for the increased risk of neutropaenic fever. However, in this trial, no patients had AST/ALT >2.5 times the UNL irrespective of liver metastasis at the enrollment. Seven patients had pretreatment grade 1 liver function abnormality. Four (57%) out of those seven patients experienced febrile neutropaenia and nine (39%) out of 23 patients who had normal liver function test experienced that event (*P*=0.34).

During the follow-up period, 25 patients received second-line chemotherapy: 20 because of disease progression, and five because of SD status after EDP. We administered salvage treatment in five patients in the status of SD after EDP chemotherapy. We censored these patients at the time of initiating the salvage treatment for the calculation of TTP, and the median TTP was 4.1 months (95% CI, 2.4–5.9). We calculated again when we did not censor at that time and rather considered as an event at the time of progression after salvage treatment. The median TTP was 4.4 months (95% CI, 2.6–6.3) with this calculation method.

In conclusion, combination chemotherapy with EDP is active and relatively well tolerated in metastatic or recurrent, unresectable gastric cancer.
